# Human Development Index and outcomes in older critically ill patients: A European multicentre study

**DOI:** 10.1016/j.aicoj.2026.100101

**Published:** 2026-06-19

**Authors:** Daniel Dankl, Raphael Romano Bruno, Michael Beil, Hans Flaatten, Malte Kelm, Sviri Sigal, Wojciech Szczeklik, Muhammed Elhadi, Michael Joannidis, Andreas Koköfer, Barbara Schreiber, Franz Singhartinger, Sandra Oeyen, Brian Marsh, Rui Moreno, Susannah Leaver, Dylan W. De Lange, Bertrand Guidet, Ariane Boumendil, Christian Jung, Bernhard Wernly

**Affiliations:** a Clinic of Anaesthesiology, Perioperative Medicine and Intensive Care Medicine, Paracelsus Medical University of Salzburg, 5020, Salzburg Austria; b Medical Faculty, Department of Cardiology, Pulmonology and Vascular Medicine, Heinrich-Heine-University Duesseldorf, 40225, Düsseldorf, Germany; c Department of Medical Intensive Care, Hadassah Medical Center and Faculty of Medicine, Hebrew University of Jerusalem, 91120, Jerusalem, Israel; d Department of Clinical Medicine, University of Bergen, Department of Anaestesia and Intensive Care, Haukeland University Hospital, 5021, Bergen, Norway; e Cardiovascular Research Institute Düsseldorf (CARID), Medical Faculty, Heinrich-Heine University, Duesseldorf, Germany; f Center for Intensive Care and Perioperative Medicine, Jagiellonian University Medical College, 31-008, Krakow, Poland; g Faculty of Medicine, University of Tripoli, R6XF+46G, Tripoli, Libya; h Division of Intensive Care and Emergency Medicine, Department of Internal Medicine, Medical University Innsbruck, 6020, Innsbruck, Austria; i Department of General, Visceral and Thoracic Surgery, Paracelsus Medical University/Salzburger Landeskliniken (SALK), Salzburg, Austria; j Department of Intensive Care 1K12IC, Ghent University Hospital, 9000, Ghent, Belgium; k Mater Misericordiae University Hospital, Dublin, D07 R2WY, Ireland; l Hospital de São José, ULS de São José, Lisbon, Portugal; m Faculdade de Ciências Médicas de Lisboa, Nova Medical School, Lisboa, Portugal; n Faculdade de Ciências da Saúde, Universidade da Beira Interior, Covilhã, Portugal; o General Intensive Care, St. George’s University Hospital NHS Foundation Trust, London, SW17 0QT, United Kingdom; p Department of Intensive Care Medicine, University Medical Center, University Utrecht, 3584 CX, Utrecht, Utrecht, The Netherlands; q Inserm, Service de'Réanimation, Sorbonne Université, Hôpital Saint-Antoine, Institut Pierre-Louis d'épidémiologie Et de Santé Publique, AP-HP, 184, Rue du Faubourg-Saint-Antoine, 75012, Paris, France; r AP-HP, Hôpital Saint-Antoine, Service de Réanimation, F75012, Paris, France; s Division of Cardiology, Pulmonology and Vascular Medicine, University Duesseldorf, Moorenstraße 5, 40225, Duesseldorf, Germany; t Institute of General Practice, Family Medicine and Preventive Medicine, Paracelsus Medical University of Salzburg, 5020, Salzburg, Austria; u Department of Internal Medicine 1, Paracelsus Medical University Salzburg, Austria

**Keywords:** HDI, VIP, ICU prognosis, Elderly, Frailty

## Abstract

**Background:**

Older adults represent an increasing proportion of intensive care unit admissions, but the relationship between country-level human development and outcomes after critical illness remains incompletely understood.

**Methods:**

We conducted a secondary analysis of three prospective multicentre registries, VIP1, VIP2, and COVIP, including acutely admitted older ICU patients with available Clinical Frailty Scale assessment, country-level Human Development Index (HDI), and 30-day vital status. VIP1 and VIP2 enrolled patients aged ≥80 years, whereas COVIP enrolled patients aged ≥70 years. The primary exposure was exceptionally high human development, defined as HDI ≥ 0.90 versus <0.90. The primary outcome was 30-day mortality. Associations were assessed using logistic regression with robust standard errors clustered by country, adjusting for age, sex, SOFA score, frailty, admission diagnosis, organ support modalities, and treatment-limitation decisions. Exploratory mediation analyses examined selected ICU management variables as potential pathways linking HDI to mortality.

**Results:**

Among 9920 patients included in the primary analysis, 8324 (83.9%) were treated in countries with HDI ≥ 0.90 and 1596 (16.1%) in countries with HDI < 0.90. Thirty-day mortality was lower in high-HDI countries than in lower-HDI countries (40.0% vs. 53.3%). In unadjusted analysis, HDI ≥ 0.90 was associated with lower 30-day mortality (OR 0.58; 95% CI 0.38–0.90; *P* = 0.016). This association persisted after multivariable adjustment (adjusted OR 0.49; 95% CI 0.31–0.80; *P* = 0.004) and was similar after additional adjustment for study cohort (aOR 0.49; 95% CI 0.31–0.77; *P* = 0.002) and ICU bed capacity (aOR 0.50; 95% CI 0.29–0.86; *P* = 0.013). When modelled continuously, higher HDI was associated with lower mortality after full adjustment (OR 0.33 per 0.10-unit increase; 95% CI 0.19–0.56; *P* < 0.001). Exploratory mediation analyses suggested that lower use of invasive mechanical ventilation in high-HDI countries may partially contribute to the observed association (NIE OR 0.86; 95% CI 0.83–0.89). Mediation analyses involving treatment-limitation decisions were more difficult to interpret because these decisions are closely linked to prognosis, clinical trajectory, and end-of-life practice.

**Conclusions:**

In this large European cohort of older critically ill patients, treatment in countries with exceptionally high human development was associated with lower 30-day mortality. The association persisted after adjustment for patient-level severity, frailty, treatment limitation, organ support, study cohort, and ICU bed capacity. These findings suggest that country-level development and ICU management patterns, particularly invasive ventilation practices, may contribute to outcome differences. Because this was an observational secondary analysis using country-level exposure data, causal interpretation should remain cautious.

## Introduction

The global population is experiencing unprecedented demographic shifts, with the proportion of very old adults (≥80 years) increasing substantially across all regions. Worldwide, approximately 70 million people are currently aged 80 years or older, with projections suggesting this number will increase more than fivefold by 2050 [1,2]. In many developed nations, adults over 65 years represent more than 20% of the population, and the proportion of those aged ≥80 years is growing at an accelerating rate 2,3]. These demographic trends have profound implications for critical care medicine. In the United States, approximately 50% of ICU admissions involve adults ≥65 years, and these elderly patients account for 60% of all ICU-days [4,5]. Similarly, in European registries, the median age of ICU patients has increased incrementally, with rapidly rising proportions of patients aged ≥70 years, particularly those aged ≥80 years and older, being admitted to intensive care units [6,7].

Complicating this demographic transition is the increasing prevalence of frailty, multimorbidity, and functional impairment in very old populations admitted to ICUs. Frailty represents a multidimensional syndrome characterized by diminished physiological reserves and increased vulnerability to acute stressors, and has emerged as a critical predictor of short-term and long-term outcomes in critically ill older adults [6,8,9]. In a large prospective European cohort of patients ≥75 years enrolled in the VIP1 study, the Clinical Frailty Scale (CFS) score was strongly associated with 30-day mortality, with a hazard ratio of 1.54 (95% confidence interval [CI] 1.38–1.73) for patients classified as frail compared with those classified as fit, with progressively higher mortality rates across frailty categories [6]. Additionally, rates of pre-existing disability, cognitive decline, multimorbidity, and functional limitations have been increasing in older patients admitted to ICUs over recent decades, trends that portend substantial burdens of post-critical illness disability in ICU survivors [7]. Very old patients frequently require multiple organ support interventions, including invasive mechanical ventilation, vasopressor therapy, and renal replacement therapy. However, the appropriateness and intensity of such interventions often depend on complex factors including illness severity, functional status, and shared goals of care.

Despite remarkable advances in critical care delivery, outcomes for old ICU patients vary substantially across regions and countries [10,11]. Multiple international studies have demonstrated that differences in intensive care utilization, resources, and outcomes cannot be explained solely by illness severity. Rather, comparisons between countries have revealed important variations in ICU bed availability, staffing models, the organisation of care, access to organ support technologies, and patterns of treatment-limitation decisions [[10], [11], [12]]. Differences in admission practices, timing of ICU transfer, discharge policies, and access to post-acute care facilities further contribute to observed variations in mortality and functional outcomes between healthcare systems [10]. Some countries, particularly those with limited ICU capacity, have established explicit policies restricting ICU admission for very elderly patients or those with severe frailty, whereas others routinely admit such patients with the goal of attempting intensive interventions [11,12]. These system-level differences raise important questions about whether country-level characteristics and healthcare infrastructure influence the outcomes of very old critically ill patients.

At the population level, country-level socioeconomic development and healthcare system capacity are strongly linked to population health outcomes and life expectancy [[13], [14], [15]]. The Human Development Index (HDI) is a composite indicator developed by the United Nations Development Programme that synthesizes three key dimensions of national development: population life expectancy at birth (reflecting health status), educational attainment (measured as mean and expected years of schooling), and gross national income per capita (reflecting standard of living) [13,14]. According to current UNDP classifications, countries are stratified into categories based on HDI thresholds, with "very high human development" defined as HDI of 0.8 or greater, and "high human development" defined as HDI between 0.7 and 0.799 [16]. However, within the very high development category, countries with exceptionally high development (HDI ≥ 0.9) represent the most advanced healthcare systems and highest population health indicators, distinct from those with HDI between 0.8 and 0.89 [16]. The HDI has been widely used to classify and compare countries' socioeconomic development and health capacity. Previous studies have documented associations between HDI and related socioeconomic indicators and broad measures of population health, including infectious disease burden, cancer incidence and mortality, and long-term health outcomes in ICU survivors [[17], [18], [19], [20]]. However, whether country-level human development is associated with specific patterns of critical care management and clinical outcomes in older ICU patients remains unclear. The relationship between national development indicators and intensive care utilization, organ support practices, treatment limitation decisions, and mortality in this particularly vulnerable population has not been well characterized. Additionally, it remains unknown whether associations between HDI and mortality in older ICU patients persist after accounting for individual-level clinical predictors, including frailty, organ dysfunction severity, and treatment limitation decisions. We chose HDI as the primary exposure because it is a widely validated composite indicator of national development that integrates health, education, and living standards, dimensions known to shape healthcare system capacity, staffing ratios, and care culture, and because it allows direct comparison across countries using a standardised metric that is independent of healthcare-specific variables such as bed availability.

To address these gaps, we conducted a large multinational secondary analysis using data from three prospective European registries of older critically ill patients: VIP1, VIP2, and COVIP. The primary objectives were to compare clinical characteristics, organ support utilization, treatment-limitation practices, and 30-day mortality between older ICU patients treated in countries with exceptionally high human development (HDI ≥ 0.90) versus those treated in countries below this threshold, and to assess whether country-level human development was associated with 30-day mortality after adjustment for age, illness severity, frailty, and ICU management variables. We focused on older critically ill patients because this population is particularly vulnerable to system-level differences in admission thresholds, treatment intensity, and end-of-life decision-making, making it an informative group in which to study the impact of national development on critical care outcomes. We hypothesized that patients treated in countries with exceptionally high human development (HDI ≥ 0.9) would have lower 30-day mortality than those treated in countries with lower HDI values, and that differences in organ support utilization and treatment limitation strategies would vary across HDI strata.

## Methods

### Study design and data sources

We conducted a secondary analysis of prospectively collected data from the VIP1, VIP2, and COVIP studies, three large international registries of very old critically ill patients. These registries enrolled consecutive ICU admissions of older adults in multiple European countries over three distinct time periods (2016–2017, 2018–2019, and 2020–2021). In all three studies, participating ICUs recorded standardised demographic, clinical, and outcome data using uniform case report forms. Local or national research ethics committees approved the original studies in accordance with national regulations, and the present analysis was performed on anonymized data without any additional contact with patients or relatives.

### Study population

For the present analysis, we included acute ICU admissions of older adults with available Clinical Frailty Scale assessment, data on the treating ICU's country, the country's Human Development Index, and 30-day vital status. The VIP1 and VIP2 registries enrolled patients aged ≥80 years, while the COVIP registry enrolled patients aged ≥70 years; all three registries targeted older critically ill patients and used comparable case report forms and outcome definitions. We excluded readmissions during the same hospital stay and patients from non-European centres without available UNDP development indicators (n = 668, primarily contributing to the COVIP registry). Of 12,494 patients across all three registries, 9920 remained for the primary analysis after applying these criteria and restricting to patients with complete outcome data; of these, 8324 (83.9%) were treated in countries with HDI ≥ 0.9 and 1596 (16.1%) in countries with HDI < 0.9. Country-level patient numbers and HDI values for all 25 participating European country-level clusters are presented in Supplementary Table S1. If multiple ICUs from the same country participated, all eligible admissions from these units were included. Country-level HDI values were assigned to 25 original registry country labels. For descriptive country-level reporting, UK-related labels were harmonized under the United Kingdom and assigned the same HDI value.

### Exposure and country-level variables

The main exposure was country-level human development. We obtained the 2021 Human Development Index and related indicators from the United Nations Development Programme (UNDP) Human Development Report 2021/2022. For each participating country, the following variables were extracted and merged to the patient-level dataset by country name:

• HDI (composite index of life expectancy, education, and gross national income per capita)
• Life expectancy at birth (years)
• Expected and mean years of schooling (years)
• Gross national income (GNI) per capita (purchasing power parity, 2021 US$)
• Gender Development Index (GDI)
• Gender Inequality Index (GII)
• Inequality-adjusted HDI (IHDI)

We pre-specified HDI as the primary exposure. For the main analyses, HDI was dichotomized as <0.90 versus ≥0.90 to distinguish countries with "high" versus "exceptionally high" human development, based on the observed distribution in our cohort and the upper range of the UNDP "very high development" category. Countries with an HDI < 0.90 included Croatia, Greece, Poland, Portugal, Romania, the Russian Federation, and Ukraine, while the group with an HDI ≥ 0.90 comprised Austria, Belgium, Cyprus, the Czech Republic, Denmark, France, Germany, Ireland, Italy, the Netherlands, Norway, Spain, Sweden, Switzerland, and the United Kingdom (including England and Wales). In sensitivity analyses, HDI was modelled as a continuous variable, scaled such that a 0.10-unit increase corresponded to an odds ratio for 30-day mortality. Additional UNDP indicators (life expectancy, education, and income) were explored in separate models as alternative continuous measures of country-level development.

The Gender Development Index (GDI) is a UNDP indicator that captures gender gaps in human development by separately calculating women’s and men’s HDI in health, education, and living standards and expressing women’s achievement as a ratio of men’s. The Gender Inequality Index (GII) is a UNDP composite measure that shows how unequal opportunities between women and men in reproductive health, empowerment (education and politics), and participation in the workforce constrain a country’s level of human development. The Inequality-adjusted Human Development Index (IHDI) modifies a country’s HDI by factoring in how unevenly health, education, and income are distributed across its population, so that greater inequality leads to a larger gap between HDI and IHDI [16,21].

### Patient-level variables

From the VIP1, VIP2, and COVIP databases, we extracted age, sex, Sequential Organ Failure Assessment (SOFA) score at ICU admission, Clinical Frailty Scale (CFS), number of chronic comorbidities, admission diagnosis, ICU length of stay, and organ support use. Organ support variables included non-invasive ventilation, endotracheal intubation, and invasive mechanical ventilation, vasopressor or inotropic therapy, and renal replacement therapy. Treatment limitation was categorized as no limitation, withholding of life-sustaining treatment, or withdrawal of life-sustaining treatment, according to the original study definitions. Information on ICU characteristics, including the total number of ICU beds and funding model (e.g., national health service vs. other), was available at the unit level and linked to each patient.

### Primary and secondary outcomes

The primary outcome was all-cause 30-day mortality after ICU admission. Vital status at day 30 was recorded in all three registries using follow-up procedures defined in the original protocols. Secondary outcomes included ICU mortality, ICU length of stay, and the frequency of treatment limitation decisions and organ support modalities across HDI strata.

### Statistical analysis

Baseline characteristics were summarized separately for patients treated in countries with HDI < 0.90 and HDI ≥ 0.90, using medians with interquartile ranges for continuous variables and counts with percentages for categorical variables. Group comparisons used Wilcoxon rank-sum tests and χ² tests as appropriate.

To evaluate the association between HDI and 30-day mortality, we fitted logistic regression models with robust standard errors clustered by country. The primary model included HDI ≥ 0.90 vs. <0.90 as the main exposure, with adjustment for age, sex, SOFA score, Clinical Frailty Scale, admission diagnosis, organ support modalities, and treatment limitation. Pre-specified sensitivity analyses additionally adjusted for ICU bed capacity per 100,000 population and study registry. To address potential temporal confounding from combining pre-pandemic and pandemic-era registries, we tested a registry-by-HDI interaction term and performed stratified analyses by registry cohort. HDI was additionally modelled as a continuous linear variable and as a restricted cubic spline with four knots, with joint significance of spline terms assessed by Wald test. Individual HDI components as life expectancy at birth, expected years of schooling, and GNI per capita were explored as alternative continuous exposures in separate unadjusted models. A sensitivity analysis excluding treatment limitation and organ support variables assessed the potential for overadjustment through mediating pathways.^1^

To explore causal mediating pathways, we conducted exploratory mediation analyses using the paramed command in Stata, sequentially examining four pre-specified mediators: any treatment limitation, invasive mechanical ventilation, renal replacement therapy, and vasopressor use. For each mediator, we estimated the natural direct effect (NDE), natural indirect effect (NIE), and marginal total effect using logistic regression for both outcome and mediator models, with all remaining organ support variables and treatment limitation included as covariates. Confidence intervals were obtained using bias-corrected bootstrap resampling with 500 replications. Admission diagnosis was entered as binary indicator variables. Because paramed does not support country-level clustered standard errors, bootstrap resampling served as the variance estimation method throughout mediation analyses.

All analyses were restricted to acute admissions with available Clinical Frailty Scale assessment and used complete-case analysis. All analyses were performed in Stata/BE 18.5.

## Results

A total of 1596 patients were treated in ICUs in countries with HDI < 0.90 and 8324 in countries with HDI ≥ 0.90. Baseline characteristics are shown in Table 1. Patients in lower-HDI countries were slightly older (median age 83 vs. 82 years), had higher illness severity (median SOFA 8 vs. 6), and higher frailty burden (CFS ≥5: 46% vs. 35%). Invasive mechanical ventilation (81% vs. 51%), vasopressor use (77% vs. 58%), and renal replacement therapy (20% vs. 10%) were substantially more frequent in lower-HDI countries, while treatment limitation was less common (20% vs. 39%). ICU bed capacity was markedly lower in lower-HDI countries (median 4 vs. 10 per 100,000 population). COVIP-era admissions accounted for 16% of patients in lower-HDI countries versus 26% in higher-HDI countries, reflecting differential registry contributions across HDI strata. Detailed patient numbers and HDI values by harmonized country are presented in Supplementary Table S1.

**Table 1:** Baseline characteristics according to Human Development Index category characteristic.

Patient characteristic	HDI < 0.90 (*N* = 1596)	HDI ≥ 0.90 (*N* = 8324)	P Value
**Demographic and clinical characteristics**			
Age – yr, median (IQR)	83 (81–86)	82 (80–86)	<0.001
SOFA score, median (IQR)	8 (5–12)	6 (3–9)	<0.001
Frailty score (CFS), median (IQR)	4 (3–6)	4 (3–5)	<0.001
Frailty category — no. (%)			<0.001
CFS ≤4	867 (54)	5426 (65)	
CFS >4	729 (46)	2898 (35)	
Male sex — no. (%)	850 (53)	4811 (58)	<0.001
Chronic comorbidity count, median (IQR)	4 (3–6)	4 (3–5)	<0.001

**Organ support and treatment limitation**			
Noninvasive ventilation — no. (%)	252 (16)	2170 (26)	<0.001
Invasive ventilation — no. (%)	1289 (81)	4263 (51)	<0.001
Vasopressor therapy — no. (%)	1229 (77)	4865 (58)	<0.001
Renal replacement therapy — no. (%)	318 (20)	839 (10)	<0.001
ICU length of stay — days, median (IQR)	6 (2–15)	4 (1–9)	<0.001

**Treatment limitation — no. (%)**			<0.001
No limitation	1282 (80)	5089 (61)	
Withhold	196 (12)	1719 (21)	
Withdraw	118 (7)	1513 (18)	
ICU mortality — no. (%)*	742 (47)	2420 (29)	<0.001
30-day mortality — no. (%)	851 (53)	3327 (40)	<0.001

**ICU admission diagnosis — no. (%)**			<0.001
COVID-19	253 (16)	2148 (26)	
Circulatory failure	134 (8)	914 (11)	
Combined respiratory and circulatory failure	277 (17)	575 (7)	
Emergency surgery	161 (10)	737 (9)	
Head injury	53 (3)	129 (2)	
Intoxication	4 (0)	32 (0)	
Multitrauma with head injury	24 (2)	105 (1)	
Multitrauma without head injury	19 (1)	119 (1)	
Non-traumatic cerebral injury	100 (6)	372 (5)	
Other	91 (6)	800 (10)	
Respiratory failure	302 (19)	1499 (18)	
Sepsis	178 (11)	816 (10)	

**Health-system and structural characteristics**			
National Health Service system — no. (%)	760 (55)	4710 (57)	0.16
ICU beds per 100,000 population, median (IQR)	4 (4–6)	10 (7–12)	<0.001

**Country-level human development indices**			
Human Development Index, 2021, median (IQR)	0.884 (0.876–0.897)	0.941 (0.915–0.958)	<0.001
Life expectancy at birth, 2021 — yr, median (IQR)	80.5 (75.6–81.4)	82.3 (81.1–83.0)	<0.001
Expected years of schooling, 2021 — yr, median (IQR)	16.8 (16.4–19.8)	17.7 (16.6–18.6)	<0.001
Mean years of schooling, 2021 — yr, median (IQR)	11.6 (9.7–13.2)	12.7 (11.8–13.5)	<0.001
GNI per capita, 2021 — 2021 PPP$, median (IQR)	38,395 (32,764–38,629)	55,020 (53,132–68,775)	<0.001
Gender Development Index, 2021, median (IQR)	0.997 (0.971–1.018)	0.979 (0.976–0.988)	<0.001
Gender Inequality Index, 2021, median (IQR)	0.087 (0.062–0.114)	0.044 (0.017–0.064)	<0.001
Inequality-adjusted HDI, 2021, median (IQR)	0.795 (0.772–0.797)	0.864 (0.827–0.887)	<0.001
HDI category — no. (%)			<0.001

**Fig. 1:**
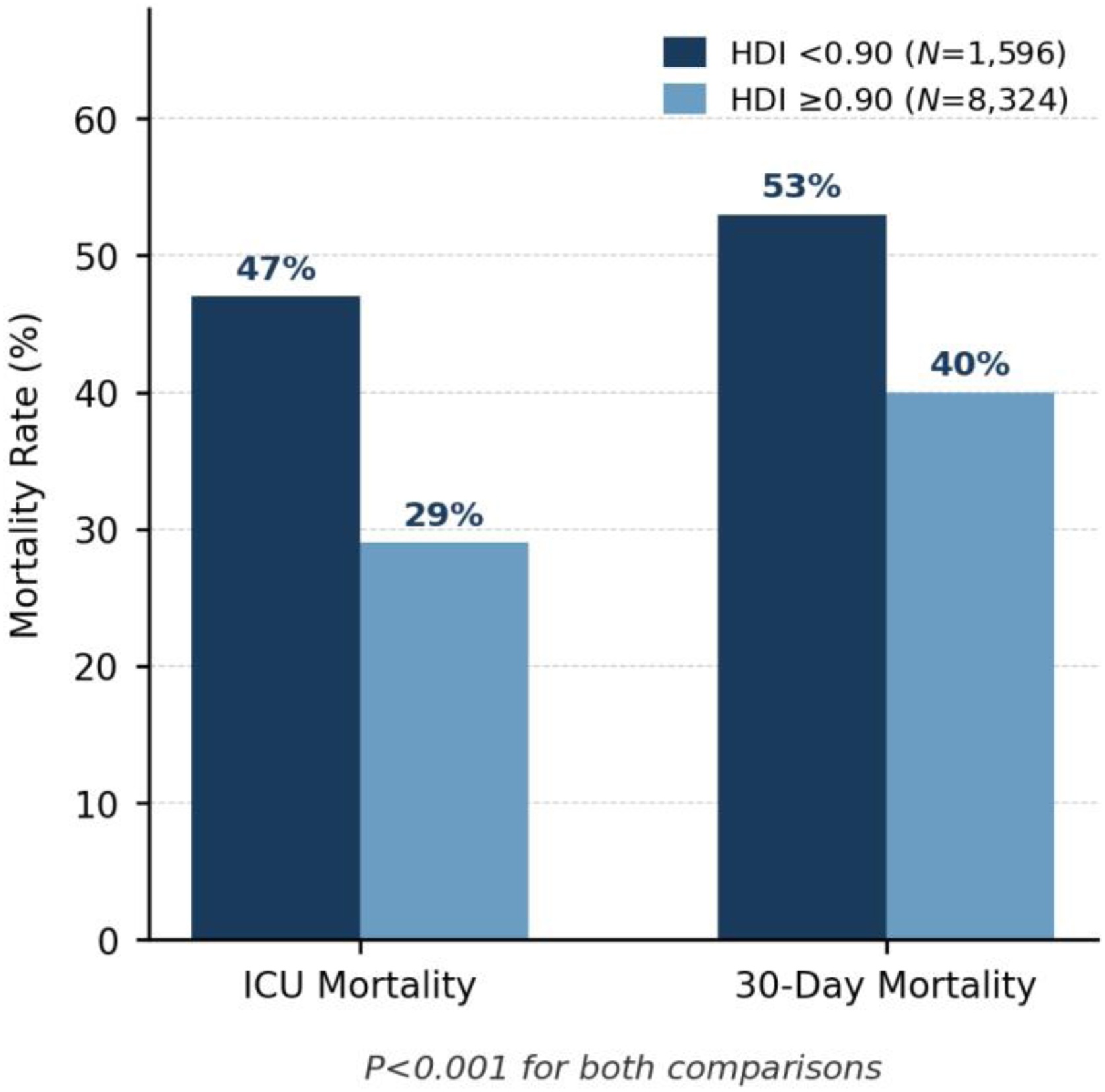
Unadjusted ICU mortality and 30-day mortality rates by country Human Development Index category (HDI < 0.90 vs. ≥0.90).

Crude 30-day mortality was 53% in lower-HDI countries and 40% in higher-HDI countries Fig.1. In unadjusted logistic regression, treatment in a country with HDI ≥ 0.90 was associated with significantly lower odds of 30-day mortality (OR 0.58; 95% CI 0.38–0.90; *P* = 0.016). After full multivariable adjustment, the association remained robust (aOR 0.49; 95% CI 0.31–0.80; *P* = 0.004). Additional adjustment for ICU bed capacity did not materially change the estimate (aOR 0.50; 95% CI 0.29–0.86; *P* = 0.013), while bed capacity itself was not independently associated with mortality (OR 1.00; 95% CI 0.97–1.03; *P* = 0.97). In the fully adjusted model, higher age, male sex, higher SOFA score, higher frailty score, invasive mechanical ventilation, renal replacement therapy, and treatment limitation orders were each independently associated with higher 30-day mortality.

To address potential temporal confounding from combining pre-pandemic and pandemic-era registries, we added study registry as a covariate to the fully adjusted model; the HDI association was essentially unchanged (aOR 0.49; 95% CI 0.31–0.77; *P* = 0.002). A registry-by-HDI interaction term was not statistically significant (*P* > 0.89 for both interaction terms), and stratified unadjusted analyses showed consistent directional associations across all three registries (COVIP: OR 0.54, *P* = 0.20; VIP1: OR 0.57, *P* = 0.011; VIP2: OR 0.55, *P* = 0.028), with attenuated precision in COVIP reflecting the smaller number of lower-HDI country clusters in that registry.

In fully adjusted models, higher HDI remained independently associated with substantially lower 30‑day mortality. When HDI was modelled as a continuous variable, higher values of HDI were associated with markedly lower odds of death (OR 0.33 per unit increase in HDI, 95% CI 0.19–0.56). This graded association was also supported by restricted cubic spline analyses. The association persisted after additional adjustment for study cohort (VIP1/VIP2) and was robust across multiple sensitivity analyses, including models with and without putative mediators and with adjustment for ICU bed capacity. In a sensitivity analysis excluding organ‑support and treatment‑limitation variables from the adjustment set, the association between HDI ≥ 0.90 and 30‑day mortality was markedly attenuated (aOR 0.88; 95% CI 0.64–1.21; P = 0.43), suggesting that ICU management variables contribute substantially to the observed HDI–mortality relationship.

Taken together, these findings are consistent with the hypothesis that the observed relationship reflects broader differences in healthcare systems, resources, and processes of care captured by the HDI, rather than being fully explained by patient‑level factors such as illness severity, frailty, or treatment intensity.In exploratory causal mediation analyses, the estimated natural direct association between HDI ≥ 0.90 and 30-day mortality was consistently below unity across mediator specifications. When invasive mechanical ventilation was specified as mediator, both the natural direct effect (NDE OR 0.52; 95% CI 0.45–0.60) and the natural indirect effect (NIE OR 0.86; 95% CI 0.83–0.89) were below unity, suggesting that lower use of invasive ventilation in high-HDI countries may partially account for the observed association. By contrast, renal replacement therapy and vasopressor use showed little evidence of meaningful mediation, with NIE estimates close to unity. The model using treatment limitation as mediator was more difficult to interpret: although the NDE remained below unity (OR 0.60; 95% CI 0.48–0.71), the indirect effect was above unity (NIE OR 2.09; 95% CI 1.94–2.26), consistent with the strong prognostic and practice-dependent nature of treatment-limitation decisions. Overall, these analyses should be interpreted as exploratory evidence that ICU management patterns, particularly invasive ventilation, may contribute to the HDI–mortality association rather than as definitive causal pathway estimates.

## Discussion

This large multinational study found that older critically ill patients treated in countries with exceptionally high human development Index ≥0.90 had lower 30-day mortality than patients treated in countries below this HDI threshold. The crude difference of approximately 13 percentage points was clinically relevant, and the association persisted in multivariable models that included age, sex, illness severity, frailty, admission diagnosis, organ support, treatment-limitation decisions, study registry, and ICU bed capacity. However, because several included management variables may reflect both clinical severity and downstream care decisions, these models should be interpreted as adjusted associations rather than definitive causal estimates. Exploratory mediation analyses suggested that invasive mechanical ventilation may partly contribute to the observed HDI–mortality association, whereas renal replacement therapy and vasopressor use showed little evidence of meaningful mediation.

Our findings align with and extend prior research on socioeconomic determinants of critical care outcomes. A recent systematic review found that lower socioeconomic status was associated with higher 30-day mortality after critical care admission, with a pooled OR of 1.13 (95% CI 1.05–1.22) [17]. A Scottish national cohort study demonstrated that patients from more deprived areas had worse long-term outcomes including longer ICU stays and more emergency readmissions, despite similar admission severity [22]. The present study extends these findings from individual-level socioeconomic status to country-level development, suggesting that system-wide factors, rather than individual disadvantage alone, independently shape outcomes in very old critically ill patients.

The pattern of care differences between HDI groups is clinically informative but should be interpreted cautiously. Lower-HDI countries used invasive mechanical ventilation more frequently and documented treatment limitations less often, while patients in these countries also had higher illness severity at admission. In exploratory mediation analysis, invasive mechanical ventilation was the only management variable with evidence of a potentially relevant indirect pathway, suggesting that lower intubation rates in high-HDI countries may partly contribute to the observed mortality difference. This finding may reflect differences in admission thresholds, early goals-of-care decisions, respiratory support strategies, staffing structures, or post-ICU care pathways. It should not be interpreted as evidence that invasive ventilation itself is causally harmful in this population, because ventilation is also a marker of acute disease severity and clinical trajectory.

The sensitivity analysis excluding organ support and treatment-limitation variables provides an important cautionary signal. In this model, the association between high HDI and 30-day mortality was substantially attenuated and no longer statistically significant. This does not invalidate the primary model, but it indicates that ICU management variables are central to the observed association and cannot be treated as simple confounders. They may simultaneously represent markers of illness severity, consequences of country-level practice patterns, and intermediates on the pathway between healthcare-system context and mortality. Accordingly, the mediation analyses should be regarded as exploratory pathway analyses rather than formal proof of causal mechanisms. This is particularly relevant for treatment limitation, which is closely linked to prognosis, clinical deterioration, family preferences, and end-of-life culture.

The substantial differences in ICU bed availability (median 4 vs. 10 per 100,000 population) suggest that lower-HDI countries operate under structural constraints that enforce stricter admission triage, consistent with the higher illness severity observed at admission. However, the HDI association was unchanged after adjusting for bed capacity, and bed availability itself carried no independent mortality signal, indicating that resource volume alone does not explain the outcome gap. This finding points toward qualitative system differences in healthcare organisation and culture as the operative mechanism rather than sheer infrastructure quantity.

The finding that individual HDI components modelled continuously were not significantly associated with mortality in unadjusted analyses, while fully adjusted continuous HDI was highly significant (OR 0.33; *P* < 0.001) and restricted cubic splines confirmed a significant overall association (χ² = 24.7; *P* < 0.001), warrants careful interpretation. The lack of significance for individual components reflects their collinearity and the limited statistical power of country-level exposures across 25 clusters, rather than absence of effect. The marginal gain in model fit from spline over dichotomous modelling was trivial (ΔPseudo-R² <0.007), supporting the dichotomous parameterisation as pragmatic and parsimonious, without implying that a strict biological threshold exists at HDI 0.90.

The robustness of findings across registry strata addresses the potential temporal confounding introduced by combining pre-pandemic and pandemic-era data. The registry-by-HDI interaction was non-significant, and directional consistency was maintained across all three cohorts, albeit with reduced precision in COVIP due to fewer lower-HDI cluster contributions in the pandemic period. The differential COVIP-era admission rates between HDI groups (16% vs. 26%) reflect this distributional imbalance rather than differential disease burden, and cohort adjustment did not materially alter the primary estimate.

Several limitations should be acknowledged. This is an observational secondary analysis, and residual confounding by unmeasured variables, including post‑ICU care quality, pre‑admission functional trajectories, and cultural attitudes toward end‑of‑life care, cannot be excluded. The mediation analyses assume no unmeasured mediator–outcome confounding and did not account for country‑level clustering; they also examined multiple mediators sequentially rather than simultaneously, so these results should be regarded as exploratory and cannot fully capture the joint contribution of management practices to the HDI–mortality relationship. Multiple exploratory and sensitivity analyses were performed without correction for multiple comparisons; therefore, these findings should be interpreted with appropriate caution and considered hypothesis‑generating rather than confirmatory. The dichotomisation of HDI at 0.90, while consistent with UNDP classifications and supported by our modelling, remains a pragmatic choice, and HDI as a country‑level exposure is ecologically defined, meaning that within‑country heterogeneity in healthcare delivery may not be captured and causal inference at the individual‑patient level requires caution. Furthermore, organ‑support and treatment‑limitation variables were measured during the ICU course and may be influenced by both baseline prognosis and country‑level care practices, so adjustment for these variables may introduce overadjustment or collider structures that cannot be fully resolved in this dataset. Finally, all participating centres were located in European countries with relatively high development and ICU capacity, which may limit the generalisability of these findings to other regions and healthcare systems.

## Conclusions

In this large European cohort of older critically ill patients, treatment in countries with exceptionally high human development was associated with lower 30-day mortality. In addition, higher HDI was consistently associated with lower 30-day mortality when modelled continuously, supporting a graded association across the HDI range rather than a simple threshold effect. This association persisted across several adjusted and sensitivity analyses, including models accounting for study registry, ICU bed capacity, and alternative HDI parameterisations. Exploratory mediation analyses suggested that ICU management patterns, particularly invasive ventilation practices, may contribute to the observed association, whereas treatment-limitation analyses were more difficult to interpret. These findings support the hypothesis that country-level development and healthcare-system context are linked to outcomes after critical illness in older adults, but causal interpretation should remain cautious because of the observational design, ecological exposure definition, and the complex role of ICU management variables.

## Declaration of Generative AI and AI-assisted technologies in the writing process

During the preparation of this work the authors used Perplexity® and ChatGPT® in order to enhance the linguistic quality and support the literature search for this manuscript. The AI tools did not contribute to the conceptualization, scientific conclusions, or interpretation of results. After using theese tools the authors reviewed and edited the content as needed and take full responsibility for the content of the published article.

## Declaration of competing interest

The authors declare the following financial interests/personal relationships which may be considered as potential competing interests:

The co-authors Michael Joannidis and Bertrand Guidet are members of the Annals of Intensive Care editorial board. If there are other authors, they declare that they have no known competing financial interests or personal relationships that could have appeared to influence the work reported in this paper.

## Footnotes

1 Multiple exploratory analyses were performed without correction for multiple comparisons.
