# Supplementary material for: Human Development Index and outcomes in older critically ill patients: A European multicentre study
Source: Ann Intensive Care. 2026 Jun 19;16:100101. doi: 10.1016/j.aicoj.2026.100101 (PMC13320540; doi:10.1016/j.aicoj.2026.100101)
Supplement: Supplementary file 1 [file mmc1.docx]

**Suppl. Table 1**

| **Country** | **n** | **HDI 2021** |
| --- | --- | --- |
| **HDI ≥0.90** |  |  |
| Austria | 131 | 0.930 |
| Belgium | 292 | 0.948 |
| Cyprus | 14 | 0.907 |
| Czech Republic | 4 | 0.901 |
| Denmark | 427 | 0.958 |
| France | 1,566 | 0.915 |
| Germany | 540 | 0.958 |
| Ireland | 188 | 0.946 |
| Italy | 600 | 0.908 |
| Netherlands | 695 | 0.951 |
| Norway | 463 | 0.969 |
| Spain | 841 | 0.912 |
| Sweden | 507 | 0.958 |
| Switzerland | 372 | 0.968 |
| United Kingdom† | 1,684 | 0.941 |
| **Total HDI ≥0.90** | **8,324** |  |
| **HDI <0.90** |  |  |
| Croatia | 3 | 0.876 |
| Greece | 448 | 0.897 |
| Poland | 630 | 0.884 |
| Portugal | 414 | 0.876 |
| Romania | 19 | 0.829 |
| Russian Federation | 45 | 0.813 |
| Ukraine | 37 | 0.772 |
| **Total HDI <0.90** | **1,596** |  |

†United Kingdom includes centres recorded separately as England (GB) (n=803), Great Britain (n=725), United Kingdom (n=145), and Wales (GB) (n=11), all assigned HDI 0.941.

668 patients from non-European centres without UNDP HDI data were excluded from all HDI-stratified analyses.
